# Investigating the Link between *STAT4* Genetic Variants, STAT4 Protein Concentrations, and Laryngeal Squamous Cell Carcinoma: A Comprehensive Analysis of Clinical Manifestations

**DOI:** 10.3390/ijms251810180

**Published:** 2024-09-22

**Authors:** Enrika Pileckaite, Alvita Vilkeviciute, Loresa Kriauciuniene, Vykintas Liutkevicius, Rasa Liutkeviciene

**Affiliations:** 1 Neuroscience Institute, Lithuanian University of Health Sciences, LT-44307 Kaunas, Lithuania; enrika.pileckaite@lsmu.lt (E.P.); loresa.kriauciuniene@lsmu.lt (L.K.); rasa.liutkeviciene@lsmu.lt (R.L.); 2 Department of Otorhinolaryngology, Lithuanian University of Health Sciences, LT-44307 Kaunas, Lithuania; vykintas.liutkevicius@lsmu.lt

**Keywords:** laryngeal squamous cell carcinoma, STAT4, single nucleotide variants, rs10181656, rs7574865, rs7601754, and rs10168266

## Abstract

According to recent research, inflammatory *STAT4* and its protein impact may be important factors in developing cancerous diseases. Still unanalyzed is this effect in patients with laryngeal squamous cell carcinoma (LSCC). In the present study, we evaluated four single nucleotide variants (SNVs) of *STAT4* (rs10181656, rs7574865, rs7601754, and rs10168266) and STAT4 serum levels to determine their link between LSCC development and its clinical manifestations. A total of 632 men (324 LSCC patients and 338 healthy individuals) were involved in this study. The genotyping was carried out using real-time PCR. Additionally, we measured 80 study subjects’ (40 LSCC patients and 40 control subjects) STAT4 protein concentrations using an enzyme-linked immunosorbent assay (ELISA). In our study, the T allele of *STAT4* rs7574865 significantly increases the likelihood of LSCC occurrence by 1.4-fold. Additionally, this SNV is associated with higher odds of early-stage disease, T1 size LSCC development, absence of metastasis to neck lymph nodes, and well-differentiated carcinoma. The G allele of rs10181656 is significantly associated with various clinical characteristics of LSCC, increasing the odds of early- and advanced-stage disease by 2.8-fold and 1.9-fold, respectively. Additionally, this allele is linked to an increased likelihood of developing tumors of different sizes and non-metastasized LSCC, as well as poorly differentiated carcinoma, highlighting its potential impact on the development and features of LSCC. Conclusion: The analysis of the *STAT4* rs7574865 SNV revealed that the G allele is linked to a more favorable prognosis in LSCC. Additionally, it is hypothesized that the G allele of rs10181656 may be associated with the occurrence of LSCC but may not serve as a sensitive prognostic biomarker for distinguishing between disease stages, cell differentiation, or tumor size.

## 1. Introduction

One of the most frequently diagnosed malignant tumors of the head and neck region is laryngeal cancer, which accounts for more than 98% of all cancer cases consisting of squamous cell carcinoma [1,2]. Laryngeal squamous cell carcinoma (LSCC) is one of the most common upper respiratory tract malignancies associated with high patient mortality and poor prognosis [3]. According to global cancer statistics, 188,960 persons had a laryngeal cancer diagnosis in 2022, and 103,216 of them patients lost their lives to the illness [4]. LSCC is more common in men than in women (4:1) [5]. Men are at a higher risk of developing LSCC than women, which may be due to the more frequent undertaking of harmful habits by men [2]. LSCC is considered to be a disease of older men, mainly affecting people over the age of sixty who have used or used to use tobacco products and alcohol [6]. Out of all detected cases of the disease, less than 10% are diagnosed in individuals younger than 40 years [7], but epidemiologic data show that LSCC is increasingly occurring in younger patients [6]. Carcinoma as a primary focus can appear in the vocal cords, laryngeal vestibule, or laryngeal lining [8]; then, the tumor cells can metastasize to the lymph nodes of the neck and spread through the blood to other regions of the head and neck and further organs [9]. Since the early symptoms of the disease (hoarseness, pain during swallowing) are not clear and specific, about 60% of patients are diagnosed at an advanced stage [1,10]. The reason behind the low five-year survival rate of LSCC patients is that the disease is often diagnosed in its later stages, and there is a substantial chance of tumor recurrence [5,11,12]. Although the exact pathogenesis of LSCC is not yet clear, it is known that the development of carcinoma is a multistep process that begins with changes in the division cycle of laryngeal epithelial cells, such as hyperplasia and dysplasia [3,8]. Currently, it is believed that the occurrence and development of LSCC is a combination of many carcinogenic factors, including long-term smoking, alcohol consumption, air pollution, gastroesophageal reflux, sex hormone metabolism disorders, and genetic predispositions [1]. Studies have shown that LSCC is a characteristic genomic imbalance that includes major chromosomal changes, such as polysomy or aneuploidy, and specific gene aberrations. Oncogene amplification, gene expression changes, and single nucleotide variants (SNVs) are molecular changes responsible for the gradual transformation of normal squamous epithelium to its malignant phenotype [13]. More and more researchers are beginning to pay attention to the possible molecular markers that can lead to the occurrence of LSCC [1]. Once biomarkers that predict disease onset are identified, they can be relied upon as potential diagnostic tools. Early diagnosis of the disease would help to detect the disease before it reaches a late stage, improve the survival rates of patients, and make it easier to apply appropriate treatment strategies [14].

The signal transducer and activator of the transcription 4 (*STAT4*) gene encodes a transcription factor that is involved not only in the regulation of gene transcription but also in the signaling pathways of the inflammatory process [15]. *STAT4* determines the functions of innate and acquired immune cells and has been identified as a susceptibility marker for autoimmune disorders [16,17]. Studies have shown that autoimmune diseases are associated with an increased risk of developing malignant tumors [18,19]. During the multistep process of tumorigenesis, cells lose their normal ability to repair DNA damage and regulate cell cycle progression and apoptosis [20]. Although *STAT4* is not known to directly contribute to cell cycle checkpoint regulation or DNA repair, *STAT4* may contribute to tumorigenesis through its close association with growth factor signaling pathways, and its involvement in apoptosis and angiogenesis processes [21]. STAT4 is involved in the manifestation of various inflammatory diseases by activating the JAK/STAT signaling pathway [22]. In this pathway, STAT4 transduces IL-12, IL-23, and type I IFN signals to T cells and monocytes, leading to T_h1_ and T_h17_ differentiation, monocyte activation, and IFNγ production [23]. STAT4 can contribute to the control of tumorigenesis by stimulating immune cells to differentiate into their inflammatory subsets, activating antitumor response cells, and activating inflammatory cytokines [23,24]. In addition, it was found that STAT4 increases the immunosuppressive activity of T cells, promotes antitumor inflammation processes, and reduces T_C_ activity, thus inhibiting the occurrence of metastases in head and neck squamous cell carcinoma [25]. Since the protein produced by *STAT4* plays an important role in inflammatory processes, mutations in *STAT4* can cause an inappropriate signaling pathway process leading to tumor development [21]. *STAT4* can also alter the tumor microenvironment by influencing the growth factors and cytokines levels, which may indirectly affect tumor cell growth and apoptosis [26]. In addition, *STAT4* SNVs are favorable prognostic markers for hepatocellular carcinoma and breast, gastric, and ovarian cancers [27].

With this study, we tried to determine the association with *STAT4* rs10181656, rs7574865, rs7601754, and rs10168266 SNVs and STAT4 protein concentration with the development of LSCC and its clinical manifestations.

## 2. Results

### 2.1. Influence of STAT4 rs10181656, rs7574865, rs7601754, and rs10168266 Variants on the Occurrence of LSCC

We analyzed the distributions of frequencies of the genotypes and alleles of *STAT4* rs10181656, rs7574865, rs7601754, and rs10168266 in the LSCC and control groups (Table 1). The distributions of the analyzed SNVs (rs10181656, rs7574865, rs7601754, and rs10168266) in the control group matched the HWE (*p* > 0.001). 

Also, we found that the distribution of *STAT4* rs10181656 CC, CG, and GG genotypes is statistically significantly different in patients with LSCC compared to the control group (38%, 46%, and 16% vs. 61.5%, 34%, and 4.4%, respectively, *p* < 0.001). The analysis showed that the G allele of *STAT4* rs10181656 is statistically significantly more frequent in patients with LSCC, compared to individuals in the control group (39% vs. 21.4%, respectively, *p* < 0.001) (Table 1).

We discovered that patients with LSCC had a statistically significant higher frequency of the T allele of *STAT4* rs7574865, as compared to those in the control group (28.1% vs. 21.3%, respectively, *p* = 0.004) (Table 1). No statistically significant difference was observed in the distribution of genotypes and alleles of the *STAT4* rs7601754 and rs10168266 variants between the individuals with LSCC and the control group (Supplementary Materials, Table S1).

We performed a binomial logistic regression analysis to evaluate the influence of selected *STAT4* variants on the manifestation of the LSCC (Table 2). The results showed that the CG genotype of *STAT4* rs10181656, compared to the CC genotype, increases the odds of developing LSCC by 2.2-fold under the codominant model (OR = 2.191, 95% CI: (1.575–3.048, *p* < 0.001), while under the overdominant model the CG genotype increases these odds by 1.7-fold (OR = 1.651, 95% CI: 1.207–2.259, *p* = 0.002). *STAT4* rs10181656 CG + GG genotypes are likely to be associated with 2.7-fold increased odds of LSCC occurrence under the dominant model (OR = 2.615, 95% CI: 1.911–3.578, *p* < 0.001), while under the codominant model, the GG genotype increases these odds by 5.9-fold (OR = 5.862, 95% CI: 3.166–10.856, *p* < 0.001), and under the recessive model, it increases these odds by 4.1-fold (OR = 4.117, 95% CI: 2.267–7.476, *p* < 0.001). According to the additive model, each G allele of rs10181656 increases the odds of LSCC development by 2.3-fold (OR = 2.316, 95% CI: 1.806–2.970, *p* < 0.001).

Analysis of *STAT4* rs7574865 revealed that compared to the GG genotype, the TT genotype increases the odds of developing LSCC by 2.4-fold under the codominant model (OR = 2.363, 95% CI: 1.227–4.550, *p* = 0.010). Each T allele was found to increase the odds of LSCC occurrence by 1.4-fold under the additive model (OR = 1.430, 95% CI: 1.114–1.836, *p* = 0.005).

Binomial logistic regression analysis of *STAT4* rs7601754 and rs10168266 variants did not reveal statistically significant results (Supplementary Materials, Table S2).

### 2.2. Associations of STAT4 rs10181656, rs7574865, rs7601754, and rs10168266 Variants with LSCC Stages

An analysis of the distribution of genotypes and alleles of *STAT4* rs10181656, rs7574865, rs7601754, and rs10168266 according to disease stages was performed. Based on the clinical information of the LSCC patients (Table 3), the stages were grouped into early (I + II) and advanced (III + IV).

The analysis revealed that the distribution of CC, CG, and GG genotypes of *STAT4* rs10181656 is statistically significantly different in both early and advanced-stage LSCC patients compared to the control group (32.6%, 49.2% and 18.2% vs. 61.5%, 34% and 4.4%, *p* < 0.001, 44.8% 42% and 13.2% vs. 61.5%, 34% and 4.4%, *p* < 0.001, respectively) (Table 4). The G allele of *STAT4* rs10181656 is statistically significantly more frequent in both early- and advanced-stage LSCC patients compared to control subjects (42.8% vs. 21.4%, *p* < 0.001, 34.3% vs. 21.4%, *p* < 0.001, respectively) (Table 4).

The results showed that the T allele of *STAT4* rs7574865 is statistically significantly more frequent in patients with an early stage of LSCC compared to the control group (29% vs. 21.3%, respectively, *p* = 0.006) (Table 4). Analysis of *STAT4* rs7601754 and rs10168266 variants did not show statistically significant differences (Supplementary Materials, Table S3).

Binomial logistic regression analysis was performed in the early-staged LSCC patients and control group (Table 5).

It revealed that the CG genotype of *STAT4* rs10181656 compared with the CC genotype increases the odds of early-stage LSCC occurrence by 2.7-fold (OR = 2.728, 95% CI: 1.829–4.071, *p* < 0.001) while under the overdominant model, CG genotype increases these odds by 1.9-fold (OR = 1.876, 95% CI: 1.298–2.711, *p* < 0.001). *STAT4* rs10181656 CG + GG genotypes were found to increase the odds of early-stage disease by 3.3-fold under the dominant model (OR = 3.308, 95% CI: 2.262–4.839, *p* < 0.001), while the GG genotype compared with CC genotype increases this odd by 7.7-fold (OR = 7.756, 95% CI: 3.948–15.238, *p* < 0.001). Under the recessive model, the GG genotype increases this odd by 4.8-fold (OR = 4.801, 95% CI: 2.530–9.111, *p* < 0.001). According to the additive model, each G allele increases the odds of early-stage LSCC development by 2.8-fold (OR = 2.763, 95% CI: 2.059–3.708, *p* < 0.001).

The analysis revealed that the TT genotype of *STAT4* rs7574865 compared with the GG genotype increases the odds of early-stage disease by 2.7-fold (OR = 2.668, 95% CI: 1.289–5.521, *p* = 0.008), and each T allele increases these odds by 1.5-fold according to the additive model (OR = 1.493, 95% CI: 1.117–1.997, *p* = 0.007).

Binomial logistic regression analysis for *STAT4* rs7601754, and rs10168266 SNVs showed no statistically significant differences (Supplementary Materials, Table S4).

A binomial logistic regression analysis was performed for patients with advanced-stage LSCC and the control group, and the results are described in Table 6. The analysis found that the CG + GG genotypes are likely to be associated with 2-times increased odds of advanced-stage LSCC occurrence under the dominant model (OR = 1.975, 95% CI: 1.329–2.934, *p* < 0.001). Also, the GG genotype of *STAT4* rs10181656 increases the odds of advanced-stage LSCC development by 4.1-fold under the codominant model (OR = 4.117, 95% CI: 1.979–8.565, *p* < 0.001), while under the recessive model, GG increases these odds by 3.3-fold (OR = 3.299, 95% CI: 1.626–6.697, *p* < 0.001). According to the additive model, each G allele of *STAT4* rs10181656 increases the odds of developing advanced-stage disease by 1.9-fold (OR = 1.886, 95% CI: 1.385–2.566, *p* < 0.001).

Binary logistic regression analysis of other *STAT4* SNVs did not yield statistically significant results (Supplementary Materials, Table S5).

### 2.3. Associations of STAT4 rs10181656, rs7574865, rs7601754, and rs10168266 Variants with LSCC Size

Based on the clinical manifestations of the LSCC, patients were divided into the following four subgroups according to the carcinomas’ size: T1, T2, T3, and T4. Frequencies of selected *STAT4* SNVs genotypes and alleles were analyzed between the subgroups of LSCC patients and control groups (Table 7 and Table 8).

The analysis revealed that the distribution of CC, CG, and GG genotypes of *STAT4* rs10181656 is statistically significantly different in all subgroups (T1, T2, T3, and T4) of LSCC size to the control group (35.9%, 47.2% and 16.2% vs. 61.5%, 34% and 4.4%, *p* < 0.001, 27.9% 55.9% and 16.2% vs. 61.5%, 34% and 4.4%, *p* < 0.001, 42.9%, 42.9% and 14.2% vs. 61.5%, 34% and 4.4%, *p* = 0.002, 46.1%, 36.8% and 17.1% vs. 61.5%, and 34% and 4.4%, *p* < 0.001, respectively). The G allele of *STAT4* rs10181656 is statistically significantly more frequent in all the LSCC subgroups (T1, T2, T3, and T4) compared to control subjects (40.2% vs. 21.4%, *p* < 0.001, 44.1% vs. 21.4%, *p* < 0.001, 35.8% vs. 21.4%, *p* < 0.001, and 35.5% vs. 21.4%, *p* < 0.001, respectively) (Table 7 and Table 8).

We also found that the *STAT4* rs7574865 T allele was more common in the T1 subgroup than in the control group (29.9% vs. 21.3%, respectively, *p* = 0.007) (Table 7).

Analysis of the T2 subgroup compared to the control group showed that the distribution of *STAT4* rs7601754 AA, AG, and GG genotypes was statistically significantly different (89.7%, 8.8%, and 1.5% vs. 70.7%, 27.5%, and 1.8%, respectively, *p* = 0.004). Also, the *STAT4* rs7601754 G allele was statistically significantly less frequent in the T2 LSCC subgroup than in the control group (5.9% vs. 15.5%, respectively, *p* = 0.003) (Table 8).

Analysis of *STAT4* rs10168266 did not yield statistically significant results (Supplementary Materials, Tables S6 and S7).

Binomial logistic regression analysis revealed that the CG genotype of *STAT4* rs10181656 compared with the CC genotype increases the odds of LSCC with T1 tumor size occurrence by 2.4-fold (OR = 2.412, 95% CI: 1.522–3.822, *p* < 0.001) while under the overdominant model, the CG genotype increases these odds by 1.8-fold (OR = 1.780, 95% CI: 1.162–2.728, *p* = 0.008). *STAT4* rs10181656 CG + GG genotypes were found to increase the odds of T1 size carcinoma by 2.9-fold under the dominant model (OR = 2.857, 95% CI: 1.846–4.422, *p* < 0.001), while the GG genotype compared with CC genotype increases this odd by 6.3-fold (OR = 6.273, 95% CI: 2.952–13.331, *p* < 0.001), and under the recessive model, the GG genotype increases this odd by 4.2-fold (OR = 4.175, 95% CI: 2.045–8.523, *p* < 0.001). According to the additive model, each G allele increases the odds of developing LSCC T1 tumor by 2.5-fold (OR = 2.471, 95% CI: 1.771–3.448, *p* < 0.001) (Table 9).

The analysis revealed that the TT genotype of *STAT4* rs7574865 compared with the GG genotype increases the odds of LSCC T1 tumor by three times (OR = 3.019, 95% CI: 1.362–6.693, *p* = 0.007), and each T allele increases these odds by 1.6-fold according to the additive model (OR = 1.557, 95% CI: 1.116–2.172, *p* = 0.009) (Table 9).

Binomial logistic regression analysis for *STAT4* rs7601754, and rs10168266 SNVs did not show any statistically significant differences (Supplementary Materials, Table S8).

The results of the binomial logistic regression analysis showed that the CG genotype of *STAT4* rs10181656 increases the odds of LSCC with T2 tumor size development by 3.6-fold (OR = 3.617, 95% CI: 1.993–6.565, *p* < 0.001) under the codominant model, while the CG genotype increases these odds by approximately 2.5-fold (OR = 2.456, 95% CI: 1.447–4.169, *p* < 0.001) under the overdominant model. The GG genotype, when compared to the CC genotype, increases the odds of T2 carcinoma occurrence by 8 times (OR = 8.028, 95% CI: 3.235–19.921, *p* < 0.001), while it increases these odds by 4.2 times under the recessive model (OR = 4.156, 95% CI: 1.817–9.505, *p* < 0.001). *STAT4* rs10181656 CG + GG genotypes were found to increase the odds of a T2 size carcinoma by 4.1-fold under the dominant model (OR = 4.126, 95% CI: 2.326–7.320, *p* < 0.001), and each G allele of the rs10181656 is associated with 3-times increased odds of developing LSCC with a T2 tumor size (OR = 3.035, 95% CI: 2.003–4.599, *p* < 0.001) (Table 10).

The analysis revealed that the *STAT4* rs7601754 AG genotype decreases the odds of developing a T2 size carcinoma by approximately four times under the codominant (OR = 0.253, 95% CI: 0.106–0.605, *p* = 0.002) and the overdominant (OR = 0.255, 95% CI: 0.107–0.609, *p* = 0.002) models, while AG + GG genotypes are associated with decreasing these odds by 3.6-fold under the dominant model (OR = 0.277, 95% CI: 0.122–0.627, *p* = 0.002). Also, each G allele of the *STAT4* rs7601754 decreases the odds of LSCC with a T2 tumor size by approximately three times under the additive model (OR = 0.332, 95% CI: 0.157–0.706, *p* = 0.004) (Table 10).

For the *STAT4* rs7574865 and rs10168266 SNVs, binomial logistic regression analysis did not reveal any statistically significant changes (Supplementary Materials, Table S9).

Moreover, the binomial logistic regression analysis showed that the *STAT4* rs10181656 GG genotype is associated with a 4.6-fold and 3.6-fold increased odds of developing LSCC T3 under the codominant (OR = 4.622, 95% CI: 1.845–11.581, *p* = 0.001) and the recessive (OR = 3.589, 95% CI: 11.496–8.611, *p* = 0.004) models. The CG + GG genotypes increase the odds of LSCC with a T3 tumor size by 2.1-fold (OR = 2.133, 95% CI: 1.237–3.679, *p* = 0.006); also, each of the G alleles increases these odds by approximately 2 times under additive model (OR = 2.025, 95% CI: 1.339–3.064, *p* < 0.001) (Table 11). Binary logistic regression analysis of other *STAT4* SNVs did not yield statistically significant results (Supplementary Materials, Table S10).

Analysis revealed that the *STAT4* rs10181656 GG genotype is associated with a 5.1-fold and 4.4-fold increased odds of developing LSCC T4 under the codominant (OR = 5.150, 95% CI: 2.258–11.747, *p* < 0.001) and the recessive (OR = 4.443, 95% CI: 2.016–9.793, *p* < 0.001) models; also, each of the G alleles increases these odds by approximately two times under the additive model (OR = 1.962, 95% CI: 1.344–2.862, *p* < 0.001) (Table 12).

Binary logistic regression analysis of *STAT4* rs7574865, rs7601754, and rs10168266 did not reveal statistically significant results in the T4 subgroup of tumor size (Supplementary Materials, Table S11).

### 2.4. Associations of STAT4 rs10181656, rs7574865, rs7601754, and rs10168266 Variants with LSCC Metastasis to the Neck Lymph Nodes

The influence of the selected SNVs on the spread of the LSCC was evaluated. LSCC patients were divided into the following groups according to the metastasis to the regional lymph nodes (Table 3): patients without metastases (N0) and patients with metastases to the neck lymph nodes (N1-N3). The distribution of CC, CG, and GG genotypes of *STAT4* rs10181656 was found to be statistically significantly different between LSCC patients without metastasis and the control group (35.9%, 45.9%, and 18.1%, vs. 61.5%, 34% and 4.5%, respectively, *p* < 0.001). The G allele of *STAT4* rs10181656 is statistically significantly more frequent in LSCC patients without lymph node metastases compared to the control group (41.1% vs. 21.4%, *p* < 0.001) (Table 13).

The results showed that the GG, GT, and TT genotypes of *STAT4* rs7574865 were statistically significantly different between LSCC patients without neck lymph node metastases and control group subjects (52.5%, 37.1%, and 10.4%, vs. 61.8%, 33.7%, and 4.5%, respectively, *p* = 0.006). The T allele of *STAT4* rs7574865 is statistically significantly more common in LSCC patients with non-metastatic tumor compared to the control group (29% vs. 21.3%, *p* = 0.002) (Table 13).

The analysis performed for *STAT4* rs7601754 and rs10168266 SNV showed no statistically significant results (Supplementary Materials, Table S12).

Binomial logistic regression analysis found that the CG genotype of *STAT4* rs10181656 increases the odds of non-metastatic to the neck lymph nodes LSCC by 2.3-fold under the codominant model (OR = 2.314, 95% CI: 1.624–3.298, *p* < 0.001), and by 1.7-fold under the overdominant model (OR = 1.648, 95% CI: 1.182–2.298, *p* = 0.003). CG + GG genotypes were found to increase the odds of non-metastasized LSCC by 2.9-fold (OR = 2.856, 95% CI: 2.042–3.994, *p* < 0.001), while only the GG genotype increases this odd by seven times and 4.8-fold under the codominant (OR = 7.008, 95% CI: 3.730–13.165, *p* < 0.001) and recessive (OR = 4.774, 95% CI: 2.603–8.755, *p* < 0.001) models, respectively. According to the additive model, each G allele of rs10181656 increases the odds of non-metastatic LSCC by 2.5-fold (OR = 2.505, 95% CI: 1.928–3.255, *p* < 0.001) (Table 14).

The analysis of *STAT4* rs7574865 showed that the TT genotype increases the odds of non-spreading to the neck lymph nodes LSCC by 2.8-fold and 2.5-fold under the codominant (OR = 2.766, 95% CI: 1.420–5.390, *p* = 0.003) and recessive (OR = 2.506, 95% CI: 1.304–4.816, *p* = 0.006) models, respectively. Each T allele of rs7574865 increased the odds of non-metastasized LSCC by 1.5 times (OR = 1.479, 95% CI: 1.140–1.920, *p* = 0.003) (Table 14).

Binomial logistic regression analysis for *STAT4* rs7601754 and rs10168266 variants did not show statistically significant results (Supplementary Materials, Table S13).

Binomial logistic regression analysis of the selected *STAT4* SNVs in LSCC patients with neck lymph node metastases and controls did not reveal any statistically significant results (Supplementary Materials, Table S14).

### 2.5. Associations of STAT4 rs10181656, rs7574865, rs7601754, and rs10168266 Variants with LSCC Differentiation Grade

Genotypes and alleles of *STAT4* rs10181656, rs7574865, rs7601754, and rs10168266 were analyzed concerning the degree of differentiation of LSCC cells. According to the clinical data of the patients (Table 3), the differentiation of carcinoma cells was divided into well (G1) and poorly (G2–G3). It was found that the distribution of CC, CG, and GG genotypes of *STAT4* rs10181656 is statistically significantly different in patients with well-differentiated and poorly differentiated LSCC compared to the control group (30.8%, 46.2% and 23.0%, vs. 61.5%, 34.0% and 4.5%, *p* < 0.001 and 40.8%, 45.9%, and 13.3%. vs. 61.5%, 34.0% and 4.5%, *p* < 0.001, respectively). The *STAT4* rs10181656 G allele is statistically significantly more frequent in patients with well-differentiated and poorly differentiated LSCC compared to the control group (46.2% vs. 21.4%, *p* < 0.001, 36.3% vs. 21.4%, *p* < 0.001, respectively) (Table 15).

*STAT4* rs7574865 GG, GT, and TT genotypes are statistically significantly different between well-differentiated LSCC patients and the control group (49.5%, 37.4%, 13.1%, vs. 61.8%, 33.7%, 4.5%, respectively, *p* = 0.004). The T allele of rs7574865 is statistically significantly more common in well-differentiated LSCC compared to healthy individuals (31.9% vs. 21.3%, *p* = 0.003) (Table 15).

No statistically significant differences were found for the *STAT4* rs10168266 and rs7601754 variants (Supplementary Materials, Table S15).

A binary logistic regression analysis of the selected polymorphisms was performed, assessing the odds of the occurrence of well-differentiated LSCC. Results showed that the *STAT4* rs10181656 CG genotype increases the odds of well-differentiated LSCC by 2.7-fold under the codominant model (OR = 2.713, 95% CI: 1.597–4.608, *p* < 0.001), while CG + GG genotypes increase these odds by 3.6-fold under the dominant model (OR = 3.600, 95% CI: 2.192–5.913, *p* < 0.001). It was found that the GG genotype increases the odds of developing well-differentiated carcinoma by 10.4-fold under the codominant model (OR = 10.400, 95% CI: 4.810–22.488, *p* < 0.001) and by 6.5-fold under the recessive (OR = 6.460, 95% CI: 3.172–13.155, *p* < 0.001) model. The additive model predicts that the likelihood of well-differentiated LSCC is increased by 3.3 times for each G allele of rs10181656 (OR = 3.086, 95% CI: 2.148–4.434, *p* < 0.001) (Table 16).

The analysis revealed that the TT genotype of *STAT4* rs7574865 increases the odds of well-differentiated LSCC by 3.7-fold and 3.3-fold under the codominant (OR = 3.716, 95% CI: 11.629–8.475, *p* = 0.002) and recessive (OR = 3.271, 95% CI: 1.473–7.265, *p* = 0.004) models, respectively. According to the additive model, each T allele of rs7574865 is associated with increased odds of well-differentiated LSCC by 1.7-fold (OR = 1.697, 95% CI: 1.185–2.432, *p* = 0.004) (Table 16).

The *STAT4* rs7601754 and rs10168266 variations did not yield statistically significant outcomes from binomial logistic regression analysis (Supplementary Materials, Table S16).

In addition, a binomial logistic regression analysis was performed for patients with poorly differentiated LSCC and the control group. It revealed that the CG genotype of *STAT4* rs10181656 increases the odds of poorly differentiated LSCC development by two times (OR = 2.037, 95% CI: 1.424–2.914, *p* < 0.001) under the codominant model, while under the overdominant model, it increases these odds by 1.7-fold (OR = 1.647, 95% CI: 1.170–2.318, *p* = 0.004). The GG genotype increases the odds of poorly differentiated carcinoma occurrence by 4.5-fold under the codominant model (OR = 4.525, 95% CI: 2.333–8.777, *p* < 0.001), while it increases these odds by 3.3-fold under the recessive model (OR = 3.305, 95% CI: 1.741–6.274, *p* < 0.001). *STAT4* rs10181656 CG + GG genotypes were found to be associated with increased odds of poorly differentiated LSCC by 2.3-fold under the dominant model (OR = 2.324, 95% CI: 1.653–3.269, *p* < 0.001), and each G allele of the rs10181656 is associated with 2.1-fold increased odds of developing LSCC with the T2 tumor size (OR = 2.087, 95% CI: 1.591–2.736, *p* < 0.001) (Table 17).

The binomial logistic regression analysis results for the *STAT4* rs7574865, rs7601754, and rs10168266 SNVs were not statistically significant (Supplementary Materials, Table S17).

### 2.6. Influence of STAT4 Protein Concentration on the Occurrence of LSCC

**Figure 1:**
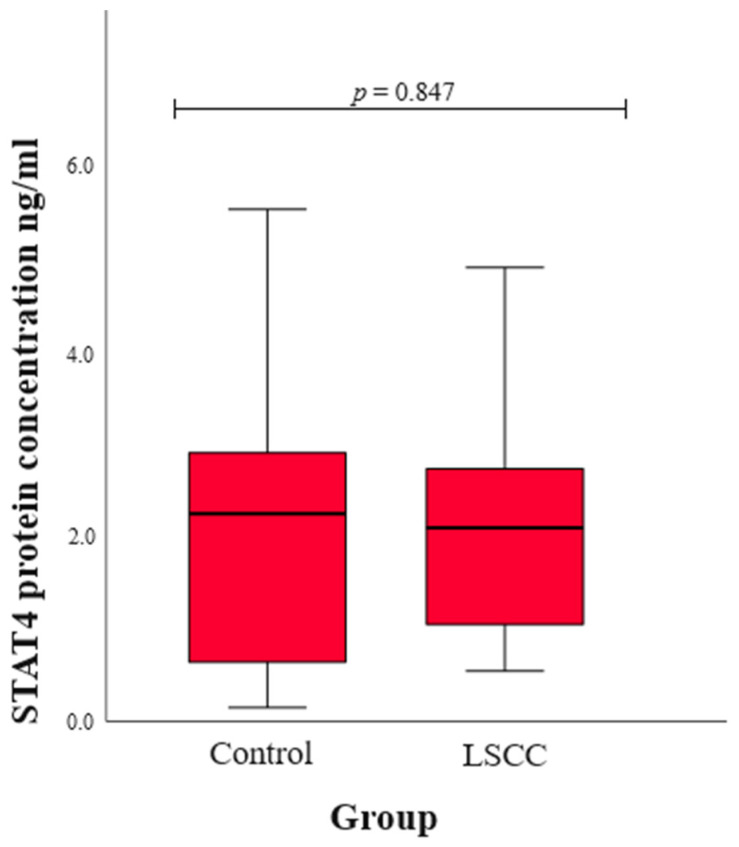
The concentration of STAT4 protein in the control and LSCC groups.

We assessed serum STAT4 levels in patients with LSCC and healthy subjects in the control group. The statistical results were compared between the LSCC and control groups, and no statistically significant difference was found (median (IQR): 2.08 (1.70) vs. 2.23 (2.29), *p* = 0.847) (Figure 1).

## 3. Discussion

STAT4, a transcription factor, regulates not only the activity of other genes but also various cellular processes, such as proliferation and survival [27]. Studies have shown that impaired *STAT4* activation is associated with the development and progression of lung, colorectal, hepatocellular, and breast cancers [26,28,29,30,31]. Based on the analysis of the literature, it can be stated that there are no studies analyzing the influence of *STAT4* rs10181656, rs7574865, rs7601754, and rs10168266 SNVs on the development of LSCC, stages of the disease, spread to neck lymph nodes, and tumor differentiation and size. In addition, there are no studies analyzing the correlation of STAT4 protein concentration with the occurrence of LSCC. The association of selected *STAT4* genetic variants with only a few cancers has been described in the literature [28,30,32,33,34].

Inflammation is not only one of the main processes in the pathogenesis of carcinogenesis [35]. It also activates the release of cytokines that promote uncontrolled cell division and enhance the proliferation of malignant tumors. Cancer is either directly or indirectly caused by mutations in growth and apoptotic proteins [36]. During tumorigenesis, specific immunoregulatory mechanisms, such as tumor-proliferating inflammation, T_h1_, T_h17_, and T_reg_ cell-mediated suppression of the immune response, may also be at play [35]. It is also known that STAT4 participates in T_h1_ and T_h17_ differentiation and plays a role in immune responses [23]. Mutations in *STAT4* can contribute to cancer by modifying immune responses, enhancing inflammation, and altering tumor cell dynamics, which highlights the importance of understanding these mutations in the context of cancer biology and potential therapeutic strategies [21,23,24,25].

*STAT4* rs7574865 is in intron 3, with a minor allele (T) occurring at a low frequency of 0.26 [37]. Although the functional significance of this SNV is not fully understood, it is hypothesized that the presence of the risk allele (T) increases *STAT4* expression, resulting in increased STAT4 phosphorylation and interferon-gamma (IFNγ) production in T lymphocytes [38]. The influence of the *STAT4* rs7574865 SNV was investigated as a cancer risk factor on the development of hepatocellular carcinoma (HCC). Yang et al. found that the GG genotype of *STAT4* rs7574865 was associated with HCC risk when comparing subjects with HCC with chronic liver disease patients (*p* = 0.030) [32]. A study by Zhong et al. found that the rs7574865 TT genotype reduced the odds of HCC compared to the GG genotype (*p* = 0.030) and that the T allele reduced the odds of HCC by 1.5-fold under the additive model (*p* = 0.044) [33]. The results of Wang et al.’s study indicated that the risk allele homozygous *STAT4* rs7574865 GG genotype may be a risk factor for the development of HCC. The results showed that the frequencies of the GG genotype and G allele are statistically significantly higher in HCC patients compared to the control group (*p* < 0.05 and *p* < 0.05, respectively). In addition, the GG genotype was found to be associated with an increased risk of HCC compared to the TT genotype (*p* < 0.05) [39]. Based on the results of the analyzed studies, the *STAT4* rs7574865 genetic variant can serve as a potential HCC prognostic biomarker [30,32,33]. In our study, each T allele of STAT4 rs7574865 increases the odds of LSCC occurrence by 1.4-fold (*p* = 0.005).Examining the influence of the *STAT4* rs7574865 variant on the clinical manifestations of LSCC, we found that the T allele increases the odds of early-stage disease by 1.5-fold, enlarges the odds of developing a T1-sized LSCC by 1.6-fold, provides 1.5-fold increased odds of the tumor not metastasizing to the neck lymph nodes, and raises the odds of a well-differentiated LSCC by 1.7-fold (*p* = 0.007, *p* = 0.009, *p* = 0.003, and *p* = 0.004, respectively). After analyzing the results of *STAT4* rs7574865, we can assume that the *STAT4* rs7574865 variant shows a significant association with various clinical manifestations of LSCC. These findings suggest a potential role of the rs7574865 T allele in a better prognosis and more favorable outcome of LSCC.

The influence of *STAT4* rs10181656, which is in the third intron, on cancer has not yet been analyzed in the literature. Our study found that each G allele of rs10181656 increases the odds of LSCC development by 2.3-fold under the additive model (*p* < 0.001). When examining the associations of this SNV with the clinical characteristics of LSCC, we observed that the G allele of rs10181656 is associated with various phenotypes of this disease. According to the additive model, each G allele of rs10181656 increases the odds of developing early- and advanced-stage LSCC by 2.8-fold and 1.9-fold (*p* < 0.001 and *p* < 0.001, respectively). However, each G allele is associated with enlarged odds of developing various sizes of tumors, such as T1, T2, T3, and T4 (*p* < 0.001, *p* < 0.001, *p* < 0.001, and *p* < 0.001, respectively). We found that each G allele of rs10181656 increases the odds of non-metastasized LSCC by 2.5-fold (*p* < 0.001) and is associated with 3.1-fold and 2.1-fold odds of well- and poorly differentiated carcinoma (*p* < 0.001 and *p* < 0.001, respectively). These findings underscore the potential impact of this SNV on the development and characteristics of LSCC. Based on the obtained results, we can assume that the G allele of rs10181656 is strongly related to the occurrence of LSCC, but, nonetheless, it is not a sensitive predictive tool to determine the specific clinical manifestations of the disease phenotype and outcomes.

Our study did not find any statistically significant results between *STAT4* rs7601754 and LSCC development. When analyzing the LSCC clinical manifestations, we found that the *STAT4* rs7601754 G allele was statistically significantly less common in the LSCC patients with T2-sized carcinoma than in the control group (*p* = 0.003). Binomial logistic regression analysis revealed that according to the most suitable model of inheritance, the AG genotype of rs7601754 reduces the odds of developing T2-sized carcinoma under the overdominant model by four times (*p* = 0.002).

Further examining studies of *STAT4* rs10168266 in the third intron, Núñez-Marrero et al. found that the T allele of *STAT4* rs10168266 was associated with a reduced risk of breast cancer (*p* = 0.03) [28]. Slattery and co-authors, examining the JAK/STAT/SOCS signaling pathways in colorectal cancer, found that *STAT4* rs10168266 CT + TT genotypes increased the risk of colon cancer under the dominant model (*p* < 0.001) [34]. Meanwhile, our study did not find any statistically significant results between *STAT4* rs10168266 and LSCC or its clinical characteristics.

Also, we assessed serum STAT4 levels in patients with LSCC and healthy controls, and the statistical comparison between the two groups revealed no significant difference. Although STAT4 serum levels do not differ between groups, the *STAT4* SNPs might still impact LSCC development through mechanisms other than altering protein levels. The SNPs could affect STAT4’s function, such as its DNA binding or interaction with other proteins, or influence post-translational modifications, thereby altering its activity [40]. They might also affect gene expression in specific cells or tissues, or lead to alternative splicing that produces functionally distinct protein isoforms. Additionally, the SNPs could influence immune responses or downstream signaling pathways, contributing to disease without changing overall STAT4 serum levels [41]. Environmental triggers and epigenetic changes might also play a role, leading to disease susceptibility. Therefore, the SNPs’ impact is likely through complex regulatory and functional changes rather than through changes in STAT4 protein levels alone [42].

## 4. Materials and Methods

The study was conducted at the Ophthalmology Laboratory of the Institute of Neurosciences of the Lithuanian University of Health Sciences (LUHS).

### 4.1. Ethics Statement

To carry out the research, we sought the permission of the Kaunas Regional Ethics Committee for Biomedical Research No. BE-2-37, date of issue: 25 March 2019. All procedure-related research was completed following the Helsinki Declaration. Before the start of the trial, all participants were made aware of its goals and design. Each participant in the research completed an informed consent form.

### 4.2. Study Population

The case–control study involved 632 individuals divided into two groups: control (*n* = 338) and patients with LSCC (*n* = 324). The LSCC patient group consisted of 324 men with an average age of 62.2 years. The control group consisted of 338 men with an average age of 61.7 years. The clinical characteristics of the LSCC patients are presented in Table 3. Data on age was compared between the LSCC and control groups, and we did not find statistically significant differences (*p* = 0.067).

### 4.3. Selection of the Study

The subjects were divided into two groups.

First group: patients with LSCC. LUHS Hospital Kaunas Clinics, Department of Ear, Nose and Throat Diseases performed the otorhinolaryngological examination for all patients with suspected LSCC. Every patient had a direct microlaryngoscopy along with a biopsy. The Department of Pathology at LSMU Hospital verified the histopathological diagnosis of LSCC. Laryngeal and neck computed tomography (CT) scans and/or magnetic resonance imaging (MRI) were carried out to obtain the final diagnosis, including staging. The National Comprehensive Cancer Network (NCCN)-accepted Guidelines for Head and Neck Cancers Classification, Version 2.2020, were used in the staging of LSCC [43]. Only men (*n* = 324) aged between 24 and 83 were included in this group.

Second group: healthy individuals as a control group (*n* = 338) aged 26 to 83 years. The group consisted only of men aged ≥18 years, in good health, free from chronic inflammatory and non-inflammatory diseases, and free from cancer.

Individuals with different types and locations of cancer, acute or chronic infectious diseases, people taking psychomotor suppressants and antiepileptic medications, and people under the age of eighteen were excluded from these groups.

### 4.4. SNV Selection

One member of the transcription factors family is STAT4, which consists of 27 exons, and which is found on human chromosome 2q32.2-q32.3 [15]. STAT4 is expressed by lymphocytes, macrophages, and dendritic cells and has a crucial role in an inflammatory process [15,21]. Based on earlier studies on inflammatory diseases and different malignant disorders [23,26,28,39,44], the *STAT4* rs7574865, rs10181656, rs7601754, and rs10168266 were selected for this investigation. Also, the selected SNVs were chosen according to the linkage disequilibrium (LD) indicators D’ (linkage disequilibrium coefficient) and r^2^ (squared correlation coefficient) applied for the SNVs that were previously mentioned in the references [23,26,28,39,44]. The LD for all SNVs was determined theoretically using the LDlink tool (accessed on 18th July 2024) [45] (Table 18 and Table 19). The selection of the SNVs were based on certain LD measures, such as a high D’ value (representing strong linkage) and a low r^2^ value (representing weak correlation). The D’ values of selected the SNVs (rs10168266, rs7601754, rs7574865, and rs10181656) are very close to 1, indicating that the alleles at these loci are inherited together almost perfectly and there has been little to no recombination between them. Their high D’ values suggest they are likely to be part of the same haplotype block or region of interest in the genome. The r^2^ values of the SNVs, namely rs10168266, rs7601754, rs7574865, and rs10181656, are not very close to 1; this indicates that while the alleles may be linked, they do not have a perfect correlation in allele frequencies, making them more interesting for further investigation. The primer sequences of chosen SNVs used are listed in Table 20.

### 4.5. Deoxyribonucleic Acid (DNA) Extraction

For DNA extraction, the blood was collected in vacuum tubes containing the anticoagulant ethylenediaminetetraacetate (EDTA). Before DNA extraction, the tube with EDTA was stored in a freezer at −80 °C. The DNA used for the study was isolated from white blood cells (leucocytes) from peripheral venous blood. The salt precipitation method was used based on the previously published method [46] which was slightly modified and applied for DNA extraction. To perform DNA isolation, the following basic equipment was required: Eppendorf Research automatic pipettes (1–1000 μL) (Eppendorf AG, Hamburg, Germany), tips (1–1000 μL) (Eppendorf AG, Hamburg, Germany), Eppendorf-type 1.5 mL test tubes (Eppendorf AG, Hamburg, Germany), plastic centrifuge tubes with a capacity of 50 mL (Ratiolab GmbH, Dreieich, Germany), and an electronic scale (“KERN 440–35N” (KERN, Balingen, Germany). First, 1 mL of blood was mixed with lysis buffer I to rupture red blood cells, which were then removed by centrifugation (centrifuge: “Fisher Accuspin Micro 17R” (Thermofisher Scientific, Hamburg, Germany)). The remaining white blood cells were treated with lysis buffer II to break down their membranes and then placed in the thermal mixer (“Thermo-Shaker TS–100” (Biosan, Ryga, Latvia). Sodium chloride was added to precipitate proteins, followed by chloroform to separate phases, leaving DNA in the water phase. Ethanol was used to precipitate the DNA, which was then pelleted, washed, and dried. Finally, the DNA was dissolved in sterile water and stored at −20 °C. All of the DNA extraction steps were performed in an Airstream Class II Biohazard Safety Cabinet (ESCO, Singapore). It is recommended to perform all steps of DNA extraction at a low temperature of about 4 °C and to store the extracted DNA at −20 °C and thaw it before use.

### 4.6. Genotyping

Studies of *STAT4* gene variants were performed using the real-time polymerase chain reaction RT-PCR method. During RT-PCR reactions, the propagation of the amplifiable fragment is measured in real-time, while the amount of product is quantified after each cycle. RT-PCR is performed in three cyclically repeated steps, as follows, the repetition of which leads to an exponential increase in the amount of DNA:

- DNA denaturation—the reaction is carried out at a temperature of 90–95 °C. At this stage, the hydrogen bonds between the nitrogenous bases are broken, and the double-stranded DNA is separated.
- Primer hybridization—the reaction is carried out at a temperature of 40–60 °C. In this stage, the primers bind to their complementary fragments of the DNA being propagated by hydrogen bonds.
- Elongation—the reaction is carried out at a temperature of 60–72 °C. At this stage, the reaction is catalyzed by the enzyme Taq polymerase, which synthesizes the complementary strand of the DNA being tested by joining the mononucleotides in the PCR mixture.

The test samples were genotyped by the RT-PCR thermal cycler “StepOne Plus” (Applied Biosystems, Foster City, CA, USA). The primers used for genotyping were developed by the company “Applied Biosystems by Thermofisher Scientifics” (Foster City, CA, USA). The composition of the RT-PCR mixture is shown in Table 21. For each reaction, 1 μL of individual DNA and 9 μL of RT-PCR reaction mixture were used. The RT-PCR reaction conditions are shown in Table 22.

### 4.7. Protein Concentration Measurement

To determine the protein concentration in the blood serum, the subjects’ blood was collected in vacuum tubes with a separating gel. After blood collection, the tubes were kept for 30 min at room temperature, in a vertical position, then centrifuged for 10 min with 1900 Relative Centrifugal Force (RCF). After centrifugation (centrifuge: “Fisher Accuspin Micro 17R” (Thermofisher Scientific, Hamburg, Germany)), the blood serum obtained from the vacuum tube with the separating gel was aspirated into a sterile Eppendorf tube and stored in a −80 °C freezer until it was used for the study. The test uses enzyme-labeled antibodies or antigens that help determine the presence of a specific antigen or antibody in a sample based on the resulting color change due to enzyme activation.

The Abbexa STAT4 ELISA kit (Abbexa Ltd., Cambridge, UK), based on solid phase “sandwich” type ELISA technology, was used to determine the concentration of STAT4 protein in blood serum in groups of subjects. The STAT4 protein concentration was measured for 40 patients with LSCC and for 40 control subjects. Test sensitivity was <0.12 ng/mL, and the test measurement limits were 0.312–20 ng/mL. In this test, the antibodies bind to the STAT4 protein-specific antigens in the blood serum and remain on the bottom of the wells. The concentrations of the samples were measured in duplicate during the ELISA.

### 4.8. Statistical Analysis

Statistical data analysis was performed using the statistical program package “Statistical Package for the Social Sciences, version 29.0 for Windows” (SPSS for Windows, version 29.0, Armonk, New York, USA). The hypothesis about the normal difference of the values of the measured characteristics was tested by applying the Shapiro–Wilk test. Since the characteristics of the subjects did not meet the criteria of a normal distribution, the median and interquartile range (IQR) indicators were used for descriptive statistics. The non-parametric Mann–Whitney U test was used to compare results in different groups with the non-normal distribution of data. The distribution of the *STAT4* gene SNV in the study groups was evaluated according to the Hardy–Weinberg equilibrium (HWE) law. The χ^2^ and Fisher exact tests were used to compare the *STAT4* gene SNV distribution homogeneity. Binary logistic regression analysis was performed by creating inheritance models (dominant, recessive, codominant, overdominant, and additive) and evaluating the odds ratio (OR) of disease occurrence with a 95% confidence interval (CI). The selection of the most reliable inheritance model was based on the Akaike information criterion (AIC), the lowest value of which indicates the most appropriate genetic model. To test statistical hypotheses, we chose the significance level (*p*) criterion, the Bonferroni correction was applied to the analysis, and a statistically significant difference was determined when the *p*-value was <0.0125.

## 5. Conclusions

Based on the results obtained in our study and the analyses conducted by other researchers, we can confirm the hypothesis that the *STAT4* rs10181656 and rs7574865 SNVs are associated with inflammatory processes that can initiate carcinogenesis. The analysis of the *STAT4* rs7574865 variant showed that the G allele is associated with a better prognosis of LSCC, namely an early stage of the disease, small tumor size without spread to the surrounding structures, and well-differentiated tumor cells in LSCC. Furthermore, we can hypothesize that the G allele of rs10181656 is associated with LSCC but that it is not a sensitive prognostic biomarker for differential analysis related to disease stages, the degree of cell differentiation, or size. Considering the obtained results, we believe that to evaluate the prognostic value of *STAT4* variants for LSCC and its clinical manifestations, it is appropriate to carry out further studies, including larger study samples which would improve the statistical power of the study, leading to more robust and reliable conclusions. Additionally, future research should incorporate environmental factors that may interact with *STAT4* variants and influence LSCC progression. This approach will provide a more comprehensive understanding of the pathological mechanisms linking *STAT4* to LSCC and will help to identify potential synergistic effects between genetic and environmental factors.

## Figures and Tables

**Table 1:** Genotype and allele frequencies of *STAT4* rs10181656 and rs7574865 in patients with LSCC and controls.

Genotype/Allele	LSCC Group, *n* (%)	Control Group, *n* (%)	HWE *p-*Value	*p*-Value
*STAT4* rs10181656				
CC	123 (38.0)	208 (61.5)	0.859	<0.001
CG	149 (46.0)	115 (34.0)		
GG	52 (16.0)	15 (4.4)		
C	395 (61.0)	531 (78.6)		<0.001
G	253 (39.0)	145 (21.4)		
*STAT4* rs7574865				
GG	171 (52.8)	209 (61.8)	0.913	0.015
GT	124 (38.3)	114 (33.7)		
TT	29 (9.0)	15 (4.4)		
G	466 (71.9)	532 (78.7)		0.004
T	182 (28.1)	144 (21.3)		

HWE—Hardy–Weinberg equilibrium; LSCC—laryngeal squamous cell carcinoma; *p—*significance level (*p* < 0.0125).

**Table 2:** Binomial logistic regression analysis of *STAT4* rs10181656 and rs7574865 in the control and patients with LSCC groups.

Model	Genotype/Allele	OR (95% CI)	*p*-Value	AIC
*STAT4* rs10181656				
Codominant	CG vs. CC GG vs. CC	2.191 (1.575–3.048) 5.862 (3.166–10.856)	<0.001 <0.001	873.638
Dominant	CG + GG vs. CC	2.615 (1.911–3.578)	<0.001	882.304
Recessive	GG vs. CG + CC	4.117 (2.267–7.476)	<0.001	893.726
Overdominant	CG vs. GG + CC	1.651 (1.207–2.259)	0.002	909.533
Additive	G	2.316 (1.806–2.970)	<0.001	871.889
*STAT4* rs7574865				
Codominant	GT vs. GG TT vs. GG	1.329 (0.961–1.840) 2.363 (1.227–4.550)	0.086 0.010	912.967
Dominant	GT + TT vs. GG	1.450 (1.064–1.975)	0.019	913.875
Recessive	TT vs. GT + GG	2.117 (1.113–4.027)	0.022	913.925
Overdominant	GT vs. TT + GG	1.218 (0.887–1.674)	0.223	917.947
Additive	T	1.430 (1.114–1.836)	0.005	911.442

OR—odds ratio, CI—confidence interval, AIC—Akaike information criterion, *p*-value—significance level (*p* < 0.0125).

**Table 3:** Demographic data of the study.

Characteristic	LSCC Group *n* = 324	Control Group *n* = 338	*p*-Value
Age, median (IQR)	62 (10)	64 (10)	0.067 *
Stages, *n* (%)			
I	113 (34.9)		
II	68 (21.0)		
III	55 (17.0)		
IV	88 (27.1)		
Tumor size (T), *n* (%)			
1	117 (36.1)		
2	68 (21.0)		
3	63 (19.4)		
4	76 (23.5)		
Metastasis to the neck lymph nodes (N), *n* (%)			
0	259 (79.9)		
1	20 (6.2)		
2	41 (12.7)		
3	4 (1.2)		
Distant metastasis (M), *n* (%)			
0	320 (98.8)		
1	4 (1.2)		
Tumor differentiation grade (G), *n* (%)			
1	91 (28.1)		
2	207 (63.9)		
3	26 (8.0)		

* Mann–Whitney U test; IQR, interquartile range; LSCC, laryngeal squamous cell carcinoma; *p*-value, significance level (*p* < 0.0125).

**Table 4:** Frequencies of *STAT4* rs10181656, rs7574865 genotypes, and alleles in patients with early- and advanced-stage LSCC and control groups.

Genotype/Allele	Control Group, *n* (%)	Early-Stage LSCC *n* (%)	*p*-Value	Advanced-Stage LSCC *n* (%)	*p*-Value
*STAT4* rs10181656					
CC	208 (61.5)	59 (32.6)	<0.001	64 (44.8)	<0.001
CG	115 (34.0)	89 (49.2)		60 (42.0)	
GG	15 (4.4)	33 (18.2)		19 (13.2)	
C	531 (78.6)	207 (57.2)	<0.001	188 (65.7)	<0.001
G	145 (21.4)	155 (42.8)		98 (34.3)	
*STAT4* rs7574865					
GG	209 (61.8)	94 (51.9)	0.016	77 (53.8)	0.158
GT	114 (33.7)	69 (38.1)		55 (38.5)	
TT	15 (4.4)	18 (9.9)		11 (7.7)	
G	532 (78.7)	257 (71.0)	0.006	209 (73.1)	0.058
T	144 (21.3)	105 (29.0)		77 (26.9)	

LSCC—laryngeal squamous cell carcinoma; *p—*significance level (*p* < 0.0125).

**Table 5:** Binomial logistic regression analysis of *STAT4* rs10181656 and rs7574865 in patients with early-stage LSCC and control groups.

Model	Genotype/Allele	OR (95% CI)	*p*-Value	AIC
*STAT4* rs10181656				
Codominant	CG vs. CC GG vs. CC	2.728 (1.829–4.071) 7.756 (3.948–15.238)	<0.001 <0.001	625.131
Dominant	CG + GG vs. CC	3.308 (2.262–4.839)	<0.001	633.118
Recessive	GG vs. CG + CC	4.801 (2.530–9.111)	<0.001	647.962
Overdominant	CG vs. GG + CC	1.876 (1.298–2.711)	<0.001	661.994
Additive	G	2.763 (2.059–3.708)	<0.001	623.140
*STAT4* rs7574865				
Codominant	GT vs. GG TT vs. GG	1.346 (0.915–1.979) 2.668 (1.289–5.521)	0.131 0.008	667.274
Dominant	GT + TT vs. GG	1.500 (1.041–2.160)	0.030	667.508
Recessive	TT vs. GT + GG	2.378 (1.168–4.840)	0.017	667.543
Overdominant	GT vs. TT + GG	1.211 (0.832–1.762)	0.318	672.250
Additive	T	1.493 (1.117–1.997)	0.007	665.927

OR—odds ratio, CI—confidence interval, AIC—Akaike information criterion, *p*-value—significance level (*p* < 0.0125).

**Table 6:** Binomial logistic regression analysis of *STAT4* rs10181656 in advanced-stage LSCC and control groups.

Model	Genotype/Allele	OR (95% CI)	*p*-Value	AIC
*STAT4* rs10181656				
Codominant	CG vs. CC GG vs. CC	1.696 (1.115–2.579) 4.117 (1.979–8.565)	0.014 <0.001	572.485
Dominant	CG + GG vs. CC	1.975 (1.329–2.934)	<0.001	575.967
Recessive	GG vs. CG + CC	3.299 (1.626–6.697)	<0.001	576.556
Overdominant	CG vs. GG + CC	1.402 (0.939–2.094)	0.099	584.727
Additive	G	1.886 (1.385–2.566)	<0.001	571.022

OR—odds ratio, CI—confidence interval, AIC—Akaike information criterion, *p*-value—significance level (*p* < 0.0125).

**Table 7:** Frequencies of *STAT4* rs10181656, rs7574865, rs7601754, genotypes, and alleles in patients with T1 and T2 LSCC size and control groups.

Genotype/Allele	Control Group, *n* (%)	T1 *n* (%)	*p*-Value	T2 *n* (%)	*p*-Value
*STAT4* rs10181656					
CC	208 (61.5)	42 (35.9)	<0.001	19 (27.9)	<0.001
CG	115 (34.0)	56 (47.9)		38 (55.9)	
GG	15 (4.4)	19 (16.2)		11 (16.2)	
C	531 (78.6)	140 (59.8)	<0.001	76 (55.9)	<0.001
G	145 (21.4)	94 (40.2)		50 (44.1)	
*STAT4* rs7574865					
GG	209 (61.8)	60 (51.3)	0.016	33 (48.5)	0.046
GT	114 (33.7)	44 (37.6)		28 (41.2)	
TT	15 (4.4)	13 (11.1)		7 (10.3)	
G	532 (78.7)	164 (70.1)	0.007	94 (69.1)	0.015
T	144 (21.3)	70 (29.9)		42 (30.9)	
*STAT4* rs7601754					
AA	239 (70.7)	82 (70.1)	0.870	61 (89.7)	0.004
AG	93 (27.5)	32 (27.4)		6 (8.8)	
GG	6 (1.8)	3 (2.6)		1 (1.5)	
A	571 (84.5)	196 (83.8)	0.798	128 (94.1)	0.003
G	105 (15.5)	38 (16.2)		8 (5.9)	

LSCC—laryngeal squamous cell carcinoma; *p—*significance level (*p* < 0.0125).

**Table 8:** Frequencies of *STAT4* rs10181656 genotypes and alleles in patients with T3 and T4 LSCC size and control groups.

Genotype/Allele	Control Group, *n* (%)	T3 *n* (%)	*p*-Value	T4 *n* (%)	*p*-Value
*STAT4* rs10181656					
CC	208 (61.5)	27 (42.9)	0.002	35 (46.1)	<0.001
CG	115 (34.0)	27 (42.9)		28 (36.8)	
GG	15 (4.4)	9 (14.2)		13 (17.1)	
C	531 (78.6)	81 (64.2)	<0.001	98 (64.5)	<0.001
G	145 (21.4)	45 (35.8)		54 (35.5)	

LSCC—laryngeal squamous cell carcinoma; *p—*significance level (*p* < 0.0125).

**Table 9:** Binomial logistic regression analysis of *STAT4* rs10181656, rs7574865 in the T1 subgroup of LSCC and control group.

Model	Genotype/Allele	OR (95% CI)	*p*-Value	AIC
*STAT4* rs10181656				
Codominant	CG vs. CC GG vs. CC	2.412 (1.522–3.822) 6.273 (2.952–13.331)	<0.001 <0.001	493.288
Dominant	CG + GG vs. CC	2.857 (1.846–4.422)	<0.001	497.602
Recessive	GG vs. CG + CC	4.175 (2.045–8.523)	<0.001	505.542
Overdominant	CG vs. GG + CC	1.780 (1.162–2.728)	0.008	513.767
Additive	G	2.471 (1.771–3.448)	<0.001	491.310
*STAT4* rs7574865				
Codominant	GT vs. GG TT vs. GG	1.344 (0.856–2.111) 3.019 (1.362–6.693)	0.198 0.007	515.129
Dominant	GT + TT vs. GG	1.539 (1.007–2.351)	0.046	516.776
Recessive	TT vs. GT + GG	2.692 (1.240–5.842)	0.012	514.770
Overdominant	GT vs. TT + GG	1.184 (0.765–1.833)	0.448	520.170
Additive	T	1.557 (1.116–2.172)	0.009	514.035

OR—odds ratio, CI—confidence interval, AIC—Akaike information criterion, *p*-value—significance level (*p* < 0.0125).

**Table 10:** Binomial logistic regression analysis of *STAT4* rs10181656 and rs7601754 in the T2 subgroup of LSCC and control group.

Model	Genotype/Allele	OR (95% CI)	*p*-Value	AIC
*STAT4* rs10181656				
Codominant	CG vs. CC GG vs. CC	3.617 (1.993–6.565) 8.028 (3.235–19.921)	<0.001 <0.001	341.572
Dominant	CG + GG vs. CC	4.126 (2.326–7.320)	<0.001	342.750
Recessive	GG vs. CG + CC	4.156 (1.817–9.505)	<0.001	358.685
Overdominant	CG vs. GG + CC	2.456 (1.447–4.169)	<0.001	357.748
Additive	G	3.035 (2.003–4.599)	<0.001	340.244
		*STAT4* rs7601754		
Codominant	AG vs. AA GG vs. AA	0.253 (0.106–0.605) 0.653 (0.077–5.526)	0.002 0.696	358.004
Dominant	AG + GG vs. AA	0.277 (0.122–0.627)	0.002	356.566
Recessive	GG vs. AG + AA	0.826 (0.098–6.972)	0.860	368.894
Overdominant	AG vs. CC + AA	0.255 (0.107–0.609)	0.002	356.172
Additive	G	0.332 (0.157–0.706)	0.004	358.231

OR—odds ratio, CI—confidence interval, AIC—Akaike information criterion, *p*-value—significance level (*p* < 0.0125).

**Table 11:** Binomial logistic regression analysis of *STAT4* rs10181656 in the T3 subgroup of LSCC and control group.

Model	Genotype/Allele	OR (95% CI)	*p*-Value	AIC
*STAT4* rs10181656				
Codominant	CG vs. CC GG vs. CC	1.809 (1.013–3.230) 4.622 (1.845–11.581)	0.045 0.001	341.514
Dominant	CG + GG vs. CC	2.133 (1.237–3.679)	0.006	343.222
Recessive	GG vs. CG + CC	3.589 (1.496–8.611)	0.004	343.494
Overdominant	CG vs. GG + CC	1.454 (0.841–2.514)	0.180	348.970
Additive	G	2.025 (1.339–3.064)	<0.001	339.811

OR—odds ratio, CI—confidence interval, AIC—Akaike information criterion, *p*-value—significance level (*p* < 0.0125).

**Table 12:** Binomial logistic regression analysis of *STAT4* rs10181656 in the T4 subgroup of LSCC and control group.

Model	Genotype/Allele	OR (95% CI)	*p*-Value	AIC
*STAT4* rs10181656				
Codominant	CG vs. CC GG vs. CC	1.447 (0.838–2.500) 5.150 (2.258–11.747)	0.185 <0.001	384.447
Dominant	CG + GG vs. CC	1.874 (1.135–3.095)	0.014	390.715
Recessive	GG vs. CG + CC	4.443 (2.016–9.793)	<0.001	384.181
Overdominant	CG vs. GG + CC	1.131 (0.674–1.898)	0.641	396.550
Additive	G	1.962 (1.344–2.862)	<0.001	384.762

OR—odds ratio, CI—confidence interval, AIC—Akaike information criterion, *p*-value—significance level (*p* < 0.0125).

**Table 13:** Frequencies of *STAT4* rs10181656 and rs7574865 genotypes and alleles in LSCC patients with and without neck lymph node metastases and control groups.

Genotype/Allele	Control Group, *n* (%)	Without Metastasis to the Neck Lymph Nodes *n* (%)	*p*-Value	With Metastasis to the Neck Lymph Nodes *n* (%)	*p*-Value
*STAT4* rs10181656					
CC	208 (61.5)	93 (35.9)	<0.001	30 (46.2)	0.061
CG	115 (34.0)	119 (45.9)		30 (46.2)	
GG	15 (4.5)	47 (18.1)		5 (7.7)	
C	531 (78.6)	305 (58.9)	<0.001	90 (69.2)	0.021
G	145 (21.4)	213 (41.1)		40 (30.8)	
*STAT4* rs7574865					
GG	209 (61.8)	136 (52.5)	0.006	35 (53.8)	0.339
GT	114 (33.7)	96 (37.1)		28 (43.1)	
TT	15 (4.5)	27 (10.4)		2 (3.1)	
G	532 (78.7)	368 (71.0)	0.002	98 (75.4)	0.402
T	144 (21.3)	150 (29.0)		32 (24.6)	

LSCC—laryngeal squamous cell carcinoma; *p—*significance level (*p* < 0.0125).

**Table 14:** Binomial logistic regression analysis of *STAT4* rs10181656 and rs7574865 in without metastases to neck lymph nodes LSCC patients and controls.

Model	Genotype/Allele	OR (95% CI)	*p*-Value	AIC
*STAT4* rs10181656				
Codominant	CG vs. CC GG vs. CC	2.314 (1.624–3.298) 7.008 (3.730–13.165)	<0.001 <0.001	769.135
Dominant	CG + GG vs. CC	2.856 (2.042–3.994)	<0.001	780.155
Recessive	GG vs. CG + CC	4.774 (2.603–8.755)	<0.001	789.079
Overdominant	CG vs. GG + CC	1.648 (1.182–2.298)	0.003	810.402
Additive	G	2.505 (1.928–3.255)	<0.001	767.560
*STAT4* rs7574865				
Codominant	GT vs. GG TT vs. GG	1.294 (0.915–1.831) 2.766 (1.420–5.390)	0.145 0.003	811.032
Dominant	GT + TT vs. GG	1.465 (1.005–2.034)	0.022	813.911
Recessive	TT vs. GT + GG	2.506 (1.304–4.816)	0.006	811.153
Overdominant	GT vs. TT + GG	1.157 (0.825–1.623)	0.397	818.418
Additive	T	1.479 (1.140–1.920)	0.003	810.357

OR—odds ratio, CI—confidence interval, AIC—Akaike information criterion, *p*-value—significance level (*p* < 0.0125).

**Table 15:** Frequencies of *STAT4* rs10181656 and rs7574865 genotypes and alleles in LSCC patients with good and poor tumor differentiation and control groups.

Genotype/Allele	Control Group, *n* (%)	Well-Differentiated LSCC *n* (%)	*p*-Value	Poorly Differentiated *n* (%)	*p*-Value
*STAT4* rs10181656					
CC	208 (61.5)	28 (30.8)	<0.001	95 (40.8)	<0.001
CG	115 (34.0)	42 (46.2)		107 (45.9)	
GG	15 (4.5)	21 (23.0)		31 (13.3)	
C	531 (78.6)	98 (53.8)	<0.001	297 (63.7)	<0.001
G	145 (21.4)	84 (46.2)		169 (36.3)	
*STAT4* rs7574865					
GG	209 (61.8)	45 (49.5)	0.004	126 (54.1)	0.114
GT	114 (33.7)	34 (37.4)		90 (38.6)	
TT	15 (4.5)	12 (13.1)		17 (7.3)	
G	532 (78.7)	124 (68.1)	0.003	342 (73.4)	0.038
T	144 (21.3)	58 (31.9)		124 (26.6)	

LSCC—laryngeal squamous cell carcinoma; *p—*significance level (*p* < 0.0125).

**Table 16:** Binomial logistic regression analysis of *STAT4* rs10181656 and rs7574865 in well-differentiated LSCC patients and controls.

Model	Genotype/Allele	OR (95% CI)	*p*-Value	AIC
*STAT4* rs10181656				
Codominant	CG vs. CC GG vs. CC	2.713 (1.597–4.608) 10.400 (4.810–22.488)	<0.001 <0.001	407.174
Dominant	CG + GG vs. CC	3.600 (2.192–5.913)	<0.001	417.714
Recessive	GG vs. CG + CC	6.460 (3.172–13.155)	<0.001	419.164
Overdominant	CG vs. GG + CC	1.662 (1.039–2.658)	0.034	440.922
Additive	G	3.086 (2.148–4.434)	<0.001	405.596
*STAT4* rs7574865				
Codominant	GT vs. GG TT vs. GG	1.385 (0.840–2.285) 3.716 (1.629–8.475)	0.202 0.002	437.895
Dominant	GT + TT vs. GG	1.656 (1.039–2.639)	0.034	440.879
Recessive	TT vs. GT + GG	3.271 (1.473–7.265)	0.004	437.507
Overdominant	GT vs. TT + GG	1.172 (0.725–1.896)	0.518	444.959
Additive	T	1.697 (1.185–2.432)	0.004	437.225

OR—odds ratio, CI—confidence interval, AIC—Akaike information criterion, *p*-value—significance level (*p* < 0.0125).

**Table 17:** Binomial logistic regression analysis of *STAT4* rs10181656 in poorly differentiated LSCC patients and controls.

Model	Genotype/Allele	OR (95% CI)	*p*-Value	AIC
*STAT4* rs10181656				
Codominant	CG vs. CC GG vs. CC	2.037 (1.424–2.914) 4.525 (2.333–8.777)	<0.001 <0.001	746.425
Dominant	CG + GG vs. CC	2.324 (1.653–3.269)	<0.001	750.158
Recessive	GG vs. CG + CC	3.305 (1.741–6.274)	<0.001	759.751
Overdominant	CG vs. GG + CC	1.647 (1.170–2.318)	0.004	765.968
Additive	G	2.087 (1.591–2.736)	<0.001	744.465

OR—odds ratio, CI—confidence interval, AIC—Akaike information criterion, *p*-value—significance level (*p* < 0.0125).

**Table 18:** All the SNVs D’ values.

SNV	rs925847	rs1400656	**rs10168266**	**rs7601754**	rs11889341	rs4274624	**rs7574865**	rs8179673	**rs10181656**	rs7582694	rs6752770	rs11685878
rs925847	1.0	0.816	0.556	0.15	0.285	0.276	0.282	0.274	0.271	0.271	0.286	0.283
rs1400656	0.816	1.0	0.896	0.954	0.835	0.837	0.836	0.759	0.838	0.838	0.323	0.438
**rs10168266**	0.556	0.896	1.0	1.0	0.872	0.871	0.872	0.871	0.871	0.871	0.409	0.055
**rs7601754**	0.15	0.954	1.0	1.0	1.0	1.0	1.0	0.953	0.976	0.976	0.162	0.273
rs11889341	0.285	0.835	0.872	1.0	1.0	1.0	0.994	1.0	1.0	1.0	0.508	0.209
rs4274624	0.276	0.837	0.871	1.0	1.0	1.0	0.994	1.0	1.0	1.0	0.508	0.212
**rs7574865**	0.282	0.836	0.872	1.0	0.994	0.994	1.0	1.0	1.0	1.0	0.504	0.205
rs8179673	0.274	0.759	0.871	0.953	1.0	1.0	1.0	1.0	1.0	1.0	0.509	0.215
**rs10181656**	0.271	0.838	0.871	0.976	1.0	1.0	1.0	1.0	1.0	1.0	0.507	0.211
rs7582694	0.271	0.838	0.871	0.976	1.0	1.0	1.0	1.0	1.0	1.0	0.507	0.211
rs6752770	0.286	0.323	0.409	0.162	0.508	0.508	0.504	0.509	0.507	0.507	1.0	0.253
rs11685878	0.283	0.438	0.055	0.273	0.209	0.212	0.205	0.215	0.211	0.211	0.253	1.0

Bolded SNVs were selected for our study.

**Table 19:** All the SNVs r^2^ values.

SNV	rs925847	rs1400656	**rs10168266**	**rs7601754**	rs11889341	rs4274624	**rs7574865**	rs8179673	**rs10181656**	rs7582694	rs6752770	rs11685878
rs925847	1.0	0.094	0.174	0.013	0.061	0.059	0.06	0.058	0.057	0.057	0.079	0.048
rs1400656	0.094	1.0	0.01	0.229	0.011	0.012	0.012	0.01	0.012	0.012	0.015	0.016
**rs10168266**	0.174	0.01	1.0	0.049	0.566	0.556	0.563	0.546	0.55	0.55	0.097	0.001
**rs7601754**	0.013	0.229	0.049	1.0	0.065	0.067	0.066	0.062	0.064	0.064	0.002	0.025
rs11889341	0.061	0.011	0.566	0.065	1.0	0.983	0.983	0.967	0.972	0.972	0.201	0.02
rs4274624	0.059	0.012	0.556	0.067	0.983	1.0	0.978	0.983	0.989	0.989	0.205	0.021
**rs7574865**	0.06	0.012	0.563	0.066	0.983	0.978	1.0	0.972	0.978	0.978	0.199	0.019
rs8179673	0.058	0.01	0.546	0.062	0.967	0.983	0.972	1.0	0.994	0.994	0.209	0.022
**rs10181656**	0.057	0.012	0.55	0.064	0.972	0.989	0.978	0.994	1.0	1.0	0.206	0.021
rs7582694	0.057	0.012	0.55	0.064	0.972	0.989	0.978	0.994	1.0	1.0	0.206	0.021
rs6752770	0.079	0.015	0.097	0.002	0.201	0.205	0.199	0.209	0.206	0.206	1.0	0.037
rs11685878	0.048	0.016	0.001	0.025	0.02	0.021	0.019	0.022	0.021	0.021	0.037	1.0

Bolded SNVs were selected for our study.

**Table 20:** Primer sequences of the *STAT4* genetic variants used in the study.

SNV	Primer Sequence
rs10181656	ACT AGC TGG AAT CCA ACT CTT CTC A**[C/G]**C CCT TGT ACC ACT ACC CTC CTT TGT
rs7574865	TAT GAA AAG TTG GTG ACC AAA ATG T**[G/T]**A ATA GTG GTT ATC TTA TTT CAG TGG
rs7601754	CAT GGG GGT GAA GAA AAG GAA CTA C**[G/A]**C AAA GAT GAT ACT AAG ACC TTG ATT
rs10168266	AGT AGT AGC TAT TGA CTA CAT GAT A**[C/T]**A CTG TCT ACC CAC CCG TAG TAA TAA

Bolded text indicates nucleotide substitution.

**Table 21:** RT-PCR mixture composition.

Reagents	Volume for 1 Sample, µL	Volume for 96 Samples, µL
TaqMan Universal Master Mix II (”Applied Biosystems by Thermofisher Scientific”, Vilnius, Lithuania)	5	480
Water without nucleases (“Invitrogen by ThermoFisher Scientific”, Paisley, UK)	3.5	336
Primer (20×) (“Applied Biosystems by Thermofisher Scientifics”, Foster City, CA, USA)	0.5	48
Full volume:	9	864

**Table 22:** RT-PCR program.

Steps	Cycle Quantity	RT-PCR Conditions	
DNA polymerase activation	1	95 °C	10 min
Denaturation	45	95 °C	15 s
Primer hybridization and elongation		60 °C	60 s
Incubation	1	4 °C	∞

## Data Availability

The data presented in this study are available on request from the corresponding author.

## Supplementary Materials

The following supporting information can be downloaded at: https://www.mdpi.com/article/10.3390/ijms251810180/s1.
