# Supplementary material for: Investigating the Link between STAT4 Genetic Variants, STAT4 Protein Concentrations, and Laryngeal Squamous Cell Carcinoma: A Comprehensive Analysis of Clinical Manifestations
Source: Int J Mol Sci. 2024 Sep 22;25(18):10180. doi: 10.3390/ijms251810180 (PMC11432593; doi:10.3390/ijms251810180)
Supplement: Supplementary file 1 [file ijms-25-10180-s001.zip › ijms-3175960-supplementary.pdf]

Table S1. Genotype and allele frequencies of STAT4 rs7601754, rs10168266 in patients with LSCC and controls

**Genotype/allele**	**LSCC group, *n* (%)**	**Control group, *n* (%)**	**HWE *p*-value**	***p*-value**
	STAT4 rs7601754			
AA	245 (75.6)	239 (70.7)	0.372	0.282
AG	72 (22.2)	93 (27.5)		
GG	7 (2.2)	6 (1.8)		
A	562 (86.7)	571 (84.5)		0.242
G	86 (13.3)	105 (15.5)		
	STAT4 rs10168266			
CC	210 (64.8)	234 (69.2)	0.300	0.275
CT	99 (30.6)	95 (28.1)		
TT	15 (4.6)	9 (2.7)		
C	519 (80.1)	563 (86.9)		0.133
T	129 (19.9)	113 (13.1)		

HWE – Hardy-Weinberg equilibrium; LSCC – laryngeal squamous cell carcinoma; *p* – significance level ($p < 0.0125$).

Table S2. Binomial logistic regression analysis of STAT4 rs7601754, rs10168266 in the control and patients with LSCC groups

**Model**	**Genotype/allele**	**OR (95% CI)**	***p*-value**	**AIC**
		STAT4 rs7601754		
Codominant	AG vs. AA	0.755 (0.529-1.078)	0.122	918.895
	GG vs. AA	1.138 (0.377-3.436)	0.819	
Dominant	AG+GG vs. AA	0.778 (0.551-1.099)	0.155	917.401
Recessive	GG vs. AG+AA	1.222 (0.406-3.675)	0.721	919.303
Overdominant	AG vs. CC+AA	0.753 (0.528-1.073)	0.116	916.948
Additive	G	0.831 (0.609-1.132)	0.240	918.044
		STAT4 rs10168266		
Codominant	CT vs. CC	1.161 (0.829-1.627)	0.386	918.830
	TT vs. CC	1.857 (0.796-4.333)	0.152	
Dominant	CT+TT vs. CC	1.221 (0.883-1.690)	0.227	917.970
Recessive	TT vs. CT+CC	1.775 (0.765-4.114)	0.181	917.584
Overdominant	CT vs. TT+CC	1.121 (0.802-1.567)	0.504	917.639
Additive	T	1.234 (0.935-1.628)	0.138	917.215

OR – odds ratio, CI – confidence interval, AIC – Akaike information criterion, *p*-value – significance level ($p < 0.0125$).

Table S3. Frequencies of STAT4 rs7601754, rs10168266 genotypes, and alleles in patients with early- and advanced-stage LSCC and control groups

**Genotype/allele**	**Control group, *n* (%)**	**Early staged LSCC *n* (%)**	***p*-value**	**Advanced staged LSCC *n* (%)**	***p*-value**
		STAT4 rs7601754			
AA	239 (70.7)	140 (77.3)	0.204	105 (73.4)	0.776
AG	93 (27.5)	37 (20.4)		35 (24.5)	
GG	6 (1.8)	4 (2.2)		3 (2.1)	
A	571 (84.5)	317 (87.6)	0.176	245 (85.7)	0.636
G	105 (15.5)	45 (12.4)		41 (14.3)	
		STAT4 rs10168266			
CC	234 (69.2)	113 (62.4)	0.130	97 (67.8)	0.869
CT	95 (28.1)	58 (32.0)		41 (28.7)	
TT	9 (2.7)	10 (5.5)		5 (3.5)	
C	563 (86.9)	284 (78.5)	0.056	235 (82.2)	0.674
T	113 (13.1)	78 (21.5)		51 (17.8)	

LSCC – laryngeal squamous cell carcinoma; *p* – significance level ($p < 0.0125$).

Table S4. Binomial logistic regression analysis of STAT4 rs7601754, rs10168266 in patients with early-stage LSCC and control groups

**Model**	**Genotype/allele**	**OR (95% CI)**	***p*-value**	**AIC**
		STAT4 rs7601754		
Codominant	AG vs. AA	0.679 (0.440-1.049)	0.081	671.991
	GG vs. AA	1.138 (0.316-4.102)	0.843	
Dominant	AG+GG vs. AA	0.707 (0.465-1.076)	0.105	670.556
Recessive	GG vs. AG+AA	1.250 (0.348-4.490)	0.732	671.126
Overdominant	AG vs. CC+AA	0.667 (0.439-1.044)	0.077	670.030
Additive	G	0.769 (0.527-1.123)	0.174	671.334
		STAT4 rs10168266		
Codominant	CT vs. CC	1.264 (0.851-1.879)	0.246	671.304
	TT vs. CC	2.301 (0.910-5.821)	0.078	
Dominant	CT+TT vs. CC	1.354 (0.927-1.978)	0.117	670.803
Recessive	TT vs. CT+CC	2.138 (0.853-5.360)	0.105	670.640
Overdominant	CT vs. TT+CC	1.201 (0.812-1.778)	0.360	671.548
Additive	T	1.362 (0.988-1.877)	0.059	669.701

OR – odds ratio, CI – confidence interval, AIC – Akaike information criterion, *p*-value – significance level ($p < 0.0125$).

Table S5. Binomial logistic regression analysis of STAT4 rs7574865, rs7601754, rs10168266 in advanced stage LSCC and control groups

**Model**	**Genotype/allele**	**OR (95% CI)**	***p*-value**	**AIC**
		STAT4 rs7574865		
Codominant	GT vs. GG	1.310 (0.865-1.982)	0.202	585.857
	TT vs. GG	1.990 (0.876-4.523)	0.100	
Dominant	GT+TT vs. GG	1.389 (0.935-2.062)	0.103	584.789
Recessive	TT vs. GT+GG	1.794 (0.803-4.009)	0.154	585.473
Overdominant	GT vs. TT+GG	1.228 (0.819-1.842)	0.321	586.451
Additive	T	1.360 (0.987-1.873)	0.060	583.936
		STAT4 rs7601754		
Codominant	AG vs. AA	0.857 (0.545-1.345)	0.502	588.919
	GG vs. AA	1.138 (0.279-4.637)	0.857	
Dominant	AG+GG vs. AA	0.874 (0.563-1.355)	0.546	587.065
Recessive	GG vs. AG+AA	1.186 (0.292-4.808)	0.811	587.376
Overdominant	AG vs. CC+AA	0.854 (0.544-1.339)	0.491	586.951
Additive	G	0.907 (0.610-1.350)	0.631	587.198
		STAT4 rs10168266		
Codominant	CT vs. CC	1.041 (0.673-1.610)	0.856	589.160
	TT vs. CC	1.340 (0.438-4.102)	0.608	
Dominant	CT+TT vs. CC	1.067 (0.701-1.624)	0.762	587.340
Recessive	TT vs. CT+CC	1.324 (0.436-4.024)	0.620	587.193
Overdominant	CT vs. TT+CC	1.028 (0.667-1.586)	0.900	587.416
Additive	T	1.081 (0.751-1.556)	0.674	587.256

OR – odds ratio, CI – confidence interval, AIC – Akaike information criterion, *p*-value – significance level ($p < 0.0125$).

Table S6. Frequencies of STAT4 rs10168266 genotypes, and alleles in patients with T1 and T2 LSCC size and control groups

**Genotype/allele**	**Control group, *n* (%)**	**T1 *n* (%)**	***p*-value**	**T2 *n* (%)**	***p*-value**
		STAT4 rs10168266			
CC	234 (69.2)	74 (63.2)	0.100	39 (57.4)	0.155
CT	95 (28.1)	35 (29.9)		26 (38.2)	
TT	9 (2.7)	8 (6.8)		3 (4.4)	
C	563 (86.9)	183 (78.2)	0.081	104 (76.5)	0.058
T	113 (13.1)	51 (21.8)		32 (23.5)	

LSCC – laryngeal squamous cell carcinoma; *p* – significance level ($p < 0.0125$).

Table S7. Frequencies of STAT4 rs7574865, rs7601754, rs10168266 genotypes, and alleles in patients with T3 and T4 LSCC size and control groups

Genotype/allele	Control group, *n* (%)	T3 *n* (%)	*p*-value	T4 *n* (%)	*p*-value
		STAT4 rs7574865			
GG	209 (61.8)	36 (57.1)	0.627	42 (55.3)	0.206
GT	114 (33.7)	25 (39.7)		27 (35.5)	
TT	15 (4.4)	2 (3.2)		7 (9.2)	
G	532 (78.7)	97 (77.0)	0.668	111 (73.0)	0.129
T	144 (21.3)	29 (23.0)		41 (27.0)	
		STAT4 rs7601754			
AA	239 (70.7)	47 (74.6)	0.517	55 (72.4)	0.425
AG	93 (27.5)	16 (25.4)		18 (23.7)	
GG	6 (1.8)	0 (0)		3 (3.9)	
A	571 (84.5)	110 (87.3)	0.414	128 (84.2)	0.937
G	105 (15.5)	16 (12.7)		24 (15.8)	
		STAT4 rs10168266			
CC	234 (69.2)	45 (71.4)	0.555	52 (68.4)	0.751
CT	95 (28.1)	15 (23.8)		23 (30.3)	
TT	9 (2.7)	3 (4.8)		1 (1.3)	
C	563 (86.9)	105 (83.3)	0.989	127 (83.6)	0.936
T	113 (13.1)	21 (16.7)		25 (16.3)	

LSCC – laryngeal squamous cell carcinoma; *p* – significance level ($p < 0.0125$).

Table S8. Binomial logistic regression analysis of STAT4 rs7601754, rs10168266 in T1 subgroup of LSCC and control group

Model	Genotype/allele	OR (95% CI)	*p*-value	AIC
		STAT4 rs7601754		
Codominant	AG vs. AA	1.003 (0.625-1.610)	0.990	522.479
	GG vs. AA	1.457 (0.356-5.960)	0.600	
Dominant	AG+GG vs. AA	1.030 (0.651-1.632)	0.898	520.727
Recessive	GG vs. AG+AA	1.456 (0.358-5.918)	0.599	520.479
Overdominant	AG vs. CC+AA	0.992 (0.619-1.589)	0.973	520.742
Additive	G	0.794 (0.699-1.597)	0.794	520.675
		STAT4 rs10168266		
Codominant	CT vs. CC	1.165 (0.730-1.859)	0.522	518.606
	TT vs. CC	2.811 (1.047-7.546)	0.040	
Dominant	CT+TT vs. CC	1.307 (0.841-2.032)	0.234	519.340
Recessive	TT vs. CT+CC	2.683 (1.010-7.125)	0.048	517.012
Overdominant	CT vs. TT+CC	1.092 (0.688-1.732)	0.709	520.605
Additive	T	1.375 (0.954-1.982)	0.088	517.895

OR – odds ratio, CI – confidence interval, AIC – Akaike information criterion, *p*-value – significance level ($p < 0.0125$).

Table S9. Binomial logistic regression analysis of STAT4 rs7574865, rs10168266 in T2 subgroup of LSCC and control group

**Model**	**Genotype/allele**	**OR (95% CI)**	***p*-value**	**AIC**
		STAT4 rs7574865		
Codominant	GT vs. GG	1.556 (0.895-2.704)	0.117	365.300
	TT vs. GG	2.956 (1.121-7.792)	0.028	
Dominant	GT+TT vs. GG	1.718 (1.018-2.901)	0.043	364.831
Recessive	TT vs. GT+GG	2.471 (0.967-6.313)	0.059	365.725
Overdominant	GT vs. TT+GG	1.375 (0.807-2.344)	0.241	367.572
Additive	T	1.648 (1.093-2.485)	0.017	363.395
		STAT4 rs10168266		
Codominant	CT vs. CC	1.642 (0.947-2.848)	0.077	367.343
	TT vs. CC	2.000 (0.519-7.714)	0.314	
Dominant	CT+TT vs. CC	1.673 (0.982-2.852)	0.059	365.420
Recessive	TT vs. CT+CC	1.687 (0.445-6.401)	0.442	368.384
Overdominant	CT vs. TT+CC	1.583 (0.919-2.727)	0.097	366.250
Additive	T	1.548 (0.985-2.434)	0.058	365.479

OR – odds ratio, CI – confidence interval, AIC – Akaike information criterion, *p*-value – significance level ($p < 0.0125$).

Table S10. Binomial logistic regression analysis of STAT4 rs7574865, rs7601754, rs10168266 in T3 subgroup of LSCC and control group

**Model**	**Genotype/allele**	**OR (95% CI)**	***p*-value**	**AIC**
		STAT4 rs7574865		
Codominant	GT vs. GG	1.273 (0.728-2.227)	0.397	351.809
	TT vs. GG	0.774 (0.170-3.529)	0.741	
Dominant	GT+TT vs. GG	1.215 (0.704-2.096)	0.484	350.256
Recessive	TT vs. GT+GG	0.706 (0.157-3.166)	0.649	350.519
Overdominant	GT vs. TT+GG	1.293 (0.744-2.247)	0.363	349.925
Additive	T	1.107 (0.699-1.754)	0.664	350.556
		STAT4 rs7601754		
Codominant	AG vs. AA	0.875 (0.473-1.619)	0.670	350.491
	GG vs. AA	-	-	
Dominant	AG+GG vs. AA	0.822 (0.445-1.518)	0.531	350.342
Recessive	GG vs. AG+AA	-	-	-
Overdominant	AG vs. CC+AA	0.897 (0.485-1.660)	0.729	350.621
Additive	G	0.780 (0.436-1.393)	0.401	350.008
		STAT4 rs10168266		
Codominant	CT vs. CC	0.821 (0.437-1.543)	0.540	351.650
	TT vs. CC	1.733 (0.452-6.653)	0.423	
Dominant	CT+TT vs. CC	0.900 (0.497-1.629)	0.728	350.621
Recessive	TT vs. CT+CC	1.828 (0.481-6.948)	0.376	350.033
Overdominant	CT vs. TT+CC	0.799 (0.427-1.495)	0.483	350.239
Additive	T	0.997 (0.600-1.654)	0.989	350.743

OR – odds ratio, CI – confidence interval, AIC – Akaike information criterion, *p*-value – significance level ($p < 0.0125$).

Table S11. Binomial logistic regression analysis of STAT4 rs7574865, rs7601754, rs10168266 in T4 subgroup of LSCC and control group

Model	Genotype/allele	OR (95% CI)	*p*-value	AIC
		STAT4 rs7574865		
Codominant	GT vs. GG	1.179 (0.691-2.012)	0.547	395.961
	TT vs. GG	2.322 (0.892-6.043)	0.084	
Dominant	GT+TT vs. GG	1.312 (0.793-2.168)	0.290	395.656
Recessive	TT vs. GT+GG	2.185 (0.858-5.559)	0.101	394.321
Overdominant	GT vs. TT+GG	1.083 (0.643-1.823)	0.765	396.678
Additive	T	1.358 (0.910-2.026)	0.134	394.583
		STAT4 rs7601754		
Codominant	AG vs. AA	0.841 (0.469-1.508)	0.561	397.245
	GG vs. AA	2.173 (0.527-8.958)	0.283	
Dominant	AG+GG vs. AA	0.922 (0.529-1.605)	0.773	396.683
Recessive	GG vs. AG+AA	2.274 (0.556-9.304)	0.253	395.589
Overdominant	AG vs. CC+AA	0.818 (0.458-1.461)	0.496	396.293
Additive	G	1.020 (0.626-1.661)	0.936	396.760
		STAT4 rs10168266		
Codominant	CT vs. CC	1.089 (0.631-1.880)	0.758	398.122
	TT vs. CC	0.500 (0.062-4.033)	0.515	
Dominant	CT+TT vs. CC	1.038 (0.608-1.775)	0.890	396.748
Recessive	TT vs. CT+CC	0.487 (0.061-3.906)	0.499	396.216
Overdominant	CT vs. TT+CC	1.110 (0.644-1.912)	0.707	396.626
Additive	T	0.980 (0.606-1.585)	0.935	396.760

OR – odds ratio, CI – confidence interval, AIC – Akaike information criterion, *p*-value – significance level ($p < 0.0125$).

Table S12. Frequencies of STAT4 rs7601754, rs10168266 genotypes and alleles in LSCC patients with and without neck lymph node metastases and control groups

Genotype/allele	Control group, *n* (%)	Without metastasis to the neck lymph nodes *n* (%)	*p*-value	With metastasis to the neck lymph nodes *n* (%)	*p*-value
		STAT4 rs7601754			
AA	239 (70.7)	199 (76.8)	0.212	46 (70.8)	0.778
AG	93 (27.5)	55 (21.2)		17 (26.2)	
GG	6 (1.8)	5 (1.9)		2 (3.1)	
A	571 (84.5)	453 (87.5)	0.144	109 (83.8)	0.858
G	105 (15.5)	65 (12.5)		21 (16.2)	
		STAT4 rs10168266			
CC	234 (69.2)	165 (63.7)	0.141	45 (69.2)	0.860
CT	95 (28.1)	80 (30.9)		19 (29.2)	
TT	9 (2.7)	14 (5.4)		1 (1.5)	
C	563 (86.9)	410 (79.2)	0.068	109 (83.8)	0.875

T	113 (13.1)	108 (20.8)	21 (16.2)	

LSCC – laryngeal squamous cell carcinoma; *p* – significance level ($p < 0.0125$).

Table S13. Binomial logistic regression analysis of STAT4 rs7601754, rs10168266 in without metastases to neck lymph nodes LSCC patients and controls

**Model**	**Genotype/allele**	**OR (95% CI)**	***p*-value**	**AIC**
		STAT4 rs7601754		
Codominant	AG vs. AA	0.710 (0.484-1.042)	0.080	818.002
	GG vs. AA	1.001 (0.301-3.328)	0.999	
Dominant	AG+GG vs. AA	0.728 (0.502-1.056)	0.094	816.295
Recessive	GG vs. AG+AA	1.089 (0.329-3.609)	0.889	819.114
Overdominant	AG vs. CC+AA	0.710 (0.485-1.040)	0.079	816.002
Additive	G	0.777 (0.555-1.088)	0.142	816.939
		STAT4 rs10168266		
Codominant	CT vs. CC	1.194 (0.835-1.708)	0.331	817.242
	TT vs. CC	2.206 (0.933-5.217)	0.072	
Dominant	CT+TT vs. CC	1.282 (0.910-1.806)	0.156	817.120
Recessive	TT vs. CT+CC	2.089 (0.890-4.905)	0.091	816.185
Overdominant	CT vs. TT+CC	1.143 (0.802-1.630)	0,459	818.587
Additive	T	1.304 (0.975-1.742)	0.073	815.917

Table S14. Binomial logistic regression analysis of STAT4 rs10181656, rs7574865, rs7601754, rs10168266 in with metastases to neck lymph nodes LSCC patients and controls

**Model**	**Genotype/allele**	**OR (95% CI)**	***p*-value**	**AIC**
		STAT4 rs10181656		
Codominant	CG vs. CC	1.809 (1.038-3.150)	0.036	354.653
	GG vs. CC	2.311 (0.783-6.820)	0.129	
Dominant	CG+GG vs. CC	1.867 (1.094-3.186)	0.022	352.842
Recessive	GG vs. CG+CC	1.794 (0.629-5.123)	0.275	357.003
Overdominant	CG vs. GG+CC	1.662 (0.971-2.844)	0.064	354.701
Additive	G	1.649 (1.078-2.521)	0.021	352.912
		STAT4 rs7574865		
Codominant	GT vs. GG	1.467 (0.849-2.534)	0.170	357.961
	TT vs. GG	0.796 (0.174-3.634)	0.769	
Dominant	GT+TT vs. GG	1.389 (0.814-2.371)	0.229	356.656
Recessive	TT vs. GT+GG	0.684 (0.153-3.063)	0.619	357.823
Overdominant	GT vs. TT+GG	1.487 (0.866-2.552)	0.150	356.052
Additive	T	1.214 (0.776-1.901)	0.395	357.384
		STAT4 rs7601754		
Codominant	AG vs. AA	0.950 (0.518-1.740)	0.867	359.645
	GG vs. AA	1.732 (0.339-8.849)	0.509	
Dominant	AG+GG vs. AA	0.997 (0.556-1.787)	0.992	358.093
Recessive	GG vs. AG+AA	1.757 (0.347-8.901)	0.496	357.673
Overdominant	AG vs. CC+AA	0.933 (0.511-1.704)	0.822	358.042
Additive	G	1.049 (0.623-1.767)	0.856	358.061
		STAT4 rs10168266		

Codominant	CT vs. CC	1.040 (0.578-1.870)	0.896	359.756
	TT vs. CC	0.578 (0.071-4.673)	0.607	
Dominant	CT+TT vs. CC	1.000 (0.563-1.777)	1.000	358.093
Recessive	TT vs. CT+CC	0.571 (0.071-4.587)	0.598	357.773
Overdominant	CT vs. TT+CC	1.057 (0.589-1.896)	0.854	358.060
Additive	T	0.956 (0.574-1.604)	0.873	358.068

OR – odds ratio, CI – confidence interval, AIC – Akaike information criterion, *p*-value – significance level ($p < 0.0125$).

Table S15. Frequencies of STAT4 rs7601754, rs10168266 genotypes and alleles in LSCC patients with good and poor tumor differentiation and control groups

**Genotype/allele**	**Control group, *n* (%)**	**Well-differentiated LSCC *n* (%)**	***p*-value**	**Poorly differentiated *n* (%)**	***p*-value**
		STAT4 rs7601754			
AA	239 (70.7)	70 (76.9)	0.436	175 (75.1)	0.429
AG	93 (27.5)	19 (20.9)		53 (22.7)	
GG	6 (1.8)	2 (2.2)		5 (2.1)	
A	571 (84.5)	159 (87.4)	0.330	403 (86.5)	0.345
G	105 (15.5)	23 (12.6)		63 (13.5)	
		STAT4 rs10168266			
CC	234 (69.2)	53 (58.2)	0.059	157 (67.4)	0.698
CT	95 (28.1)	32 (35.2)		67 (28.8)	
TT	9 (2.7)	6 (6.6)		9 (3.9)	
C	563 (86.9)	138 (75.8)	0.021	381 (81.8)	0.504
T	113 (13.1)	44 (24.2)		85 (18.2)	

LSCC – laryngeal squamous cell carcinoma; *p* – significance level ($p < 0.0125$).

Table S16. Binomial logistic regression analysis of STAT4 rs7601754, rs10168266 in well-differentiated LSCC patients and controls

**Model**	**Genotype/allele**	**OR (95% CI)**	***p*-value**	**AIC**
		STAT4 rs7601754		
Codominant	AG vs. AA	0.698 (0.398-1.222)	0.208	445.655
	GG vs. AA	1.138 (0.225-5.764)	0.876	
Dominant	AG+GG vs. AA	0.724 (0.422-1.244)	0.242	443.960
Recessive	GG vs. AG+AA	1.243 (0.247-6.266)	0.792	445.307
Overdominant	AG vs. CC+AA	0.695 (0.397-1.216)	0.202	443.679
Additive	G	0.781 (0.478-1.277)	0.325	444.368
		STAT4 rs10168266		
Codominant	CT vs. CC	1.487 (0.903-2.450)	0.119	442.172
	TT vs. CC	2.943 (1.004-8.626)	0.049	
Dominant	CT+TT vs. CC	1.613 (1.002-2.598)	0.049	441.567
Recessive	TT vs. CT+CC	2.580 (0.894-7.449)	0.080	442.551

Overdominant	CT vs. TT+CC	1.387 (0.849-2.268)	0.192	443.700
Additive	T	1.586 (1.066-2.358)	0.023	440.345

OR – odds ratio, CI – confidence interval, AIC – Akaike information criterion, *p*-value – significance level ($p < 0.0125$).

Table S17. Binomial logistic regression analysis of STAT4 rs7574865, rs7601754, rs10168266 in poorly differentiated LSCC patients and controls

**Model**	**Genotype/allele**	**OR (95% CI)**	***p*-value**	**AIC**
		STAT4 rs7574865		
Codominant	GT vs. GG	1.310 (0.919-1.866)	0.135	771.839
	TT vs. GG	1.880 (0.907-3.896)	0.090	
Dominant	GT+TT vs. GG	1.376 (0.981-1.930)	0.065	770.740
Recessive	TT vs. GT+GG	1.695 (0.829-3.466)	0.148	772.066
Overdominant	GT vs. TT+GG	1.237 (0.874-1.750)	0.230	772.719
Additive	T	1.339 (1.015-1.766)	0.039	769.878
		STAT4 rs7601754		
Codominant	AG vs. AA	0.778 (0.527-1.149)	0.207	774.449
	GG vs. AA	1.138 (0.342-3.789)	0.833	
Dominant	AG+GG vs. AA	0.800 (0.548-1.168)	0.248	772.808
Recessive	GG vs. AG+AA	1.213 (0.366-4.024)	0.752	774.056
Overdominant	AG vs. CC+AA	0.776 (0.526-1.144)	0.200	772.493
Additive	G	0.847 (0.603-1.192)	0.341	773.240
		STAT4 rs10168266		
Codominant	CT vs. CC	1.051 (0.724-1.525)	0.793	775.447
	TT vs. CC	1.490 (0.579-3.838)	0.408	
Dominant	CT+TT vs. CC	1.089 (0.761-1.558)	0.640	773.938
Recessive	TT vs. CT+CC	1.469 (0.574-3.758)	0.423	773.516
Overdominant	CT vs. TT+CC	1.032 (0.713-1.494)	0.866	774.127
Additive	T	1.110 (0.816-1.512)	0.506	773.714

OR – odds ratio, CI – confidence interval, AIC – Akaike information criterion, *p*-value – significance level ($p < 0.0125$).
